# Compromised word-level neural tracking in the high-gamma band for children with attention deficit hyperactivity disorder

**DOI:** 10.3389/fnhum.2023.1174720

**Published:** 2023-05-05

**Authors:** Cheng Luo, Yayue Gao, Jianing Fan, Yang Liu, Yonglin Yu, Xin Zhang

**Affiliations:** ^1^Research Center for Applied Mathematics and Machine Intelligence, Research Institute of Basic Theories, Zhejiang Lab, Hangzhou, China; ^2^Department of Psychology, School of Humanities and Social Sciences, Beihang University, Beijing, China; ^3^Department of Rehabilitation, The Children’s Hospital, Zhejiang University School of Medicine, National Clinical Research Center for Child Health, Hangzhou, China; ^4^Department of Neurology, The Children’s Hospital, Zhejiang University School of Medicine, National Clinical Research Center for Child Health, Hangzhou, China

**Keywords:** ADHD, speech perception, neural tracking, acoustic encoding, linguistic processing

## Abstract

Children with attention deficit hyperactivity disorder (ADHD) exhibit pervasive difficulties in speech perception. Given that speech processing involves both acoustic and linguistic stages, it remains unclear which stage of speech processing is impaired in children with ADHD. To investigate this issue, we measured neural tracking of speech at syllable and word levels using electroencephalography (EEG), and evaluated the relationship between neural responses and ADHD symptoms in 6–8 years old children. Twenty-three children participated in the current study, and their ADHD symptoms were assessed with SNAP-IV questionnaires. In the experiment, the children listened to hierarchical speech sequences in which syllables and words were, respectively, repeated at 2.5 and 1.25 Hz. Using frequency domain analyses, reliable neural tracking of syllables and words was observed in both the low-frequency band (<4 Hz) and the high-gamma band (70–160 Hz). However, the neural tracking of words in the high-gamma band showed an anti-correlation with the ADHD symptom scores of the children. These results indicate that ADHD prominently impairs cortical encoding of linguistic information (e.g., words) in speech perception.

## 1. Introduction

Attention deficit hyperactivity disorder (ADHD) is a common childhood mental disorder characterized by attention deficit, hyperactivity and/or impulsivity, and cognitive dysfunction (Mueller et al., 2017; Katya, 2018). Extensive evidence has demonstrated that children with ADHD exhibit difficulties in speech perception, e.g., recognizing speech in noisy environments or extracting target speech from competing speakers (Davidson and Prior, 1978; Keith et al., 1989; Cook et al., 1993; Pillsbury et al., 1995; Geffner et al., 1996; Gomez and Condon, 1999; Schafer et al., 2013; Lanzetta-Valdo et al., 2017; Blomberg et al., 2019). Given that speech perception is a complex process involving both low-level acoustic encoding and high-level linguistic processing, it remains unclear which stage of speech processing is prominently impaired in children with ADHD.

On the one hand, it has been hypothesized that children with ADHD have difficulties in neural encoding of acoustic features, especially for a complex auditory scene consisting of multiple acoustic distractors. Evidence shows that children with ADHD (vs. healthy control children) have reduced neural responses to target sounds under distractors (Loiselle et al., 1980; Jonkman et al., 1997; Gomes et al., 2012). It is also found that children with ADHD exhibit impairments in motor synchrony of acoustic rhythm (Rubia et al., 2001; Toplak and Tannock, 2005; Ben-Pazi et al., 2006; Puyjarinet et al., 2017), which is associated with the compromised neural encoding of the auditory envelope (Tierney and Kraus, 2013; Carr et al., 2014). Specifically, acoustic rhythm is carried by the auditory envelope (i.e., the temporal fluctuations of sound power), which captures acoustic information about duration, tempo, and stress (Kotz et al., 2018; Myers et al., 2019; Poeppel and Assaneo, 2020). When the envelope is corrupted in speech, neural encoding of the auditory envelope degrades (Doelling et al., 2014), and speech intelligibility drops (Ghitza, 2012). Since the auditory envelope provides important cues for syllabic boundaries in speech (Giraud and Poeppel, 2012; Ding et al., 2014; Poeppel and Assaneo, 2020), the neural encoding of the auditory envelope might reflect an intermediate neural process to link the auditory representation of acoustic speech features and phonological representation of syllables, and therefore play a critical role in speech perception (Giraud and Poeppel, 2012; Poeppel and Assaneo, 2020).

On the other hand, speech perception entails not only acoustic encoding, but also higher-level linguistic processing. Since children with ADHD usually suffer from co-occurring linguistic impairments (Seidman et al., 1998; Cohen et al., 2000; Litovsky, 2015; Mueller et al., 2017; Randell et al., 2019), it has also been hypothesized that degraded linguistic processing induces difficulties in speech perception for children with ADHD (Bellani et al., 2011; Laffere et al., 2021). This hypothesis is motivated by two sides of observations. First, ADHD children do not always show behavioral impairments in acoustic detection performance (Satterfield et al., 1990; Rothenberger et al., 2000; van Mourik et al., 2011; Michalek et al., 2014). Accordingly, electrophysiological evidence also finds comparable neural responses to target sounds (Rothenberger et al., 2000) and acoustic envelope (Laffere et al., 2021) between children with and without ADHD. Second, ADHD children exhibit significantly reduced word identification and speech discrimination ability compared with typically developing children (Norrelgen et al., 1999; Fuermaier et al., 2018). Moreover, abnormal neural activity and connectivity are also observed in ADHD children during word identification (Murias et al., 2007). All these findings suggest that ADHD might impair higher-level linguistic processing beyond low-level acoustic encoding in speech perception.

Despite converging evidence for neurocognitive deficits in speech perception for children with ADHD, few studies have analyzed the underlying neural mechanisms across multiple processing stages. One methodological challenge is the difficulty in dissociating neural representations of multiple stages in speech processing. Here, we adopted a hierarchical auditory linguistic sequence paradigm to quantify the neural responses tracking two levels of speech units, i.e., syllables and words (Ding et al., 2018; Jin et al., 2020). The rationale is that cortical activity tracks different levels of speech units during speech perception (Luo and Poeppel, 2007; Ding et al., 2016, 2018; Gao et al., 2020). When syllables and words are presented at a unique and constant rate in speech, the neural tracking responses to syllables and words are tagged at distinct frequencies (Ding et al., 2016). Neural tracking of syllables and words can reflect two stages of speech processing: Neural tracking of syllables is interconnected to acoustic encoding of speech features, i.e., speech envelope (Giraud and Poeppel, 2012; Poeppel and Assaneo, 2020), and neural tracking of words reflects higher-level linguistic processing (Martin and Doumas, 2017; Jin et al., 2020; Meyer et al., 2020; Lu et al., 2022). The locus of the ADHD effects is important because it can offer effective guidance for how the remediation should be targeted to ADHD children who have difficulty in speech perception (Laffere et al., 2021). The goal of the current study is twofold. First, we explored whether cortical activity of young children could track syllables and words in a continuous speech stream. Six-eight years old children with mild ADHD symptoms were recruited to take part in a speech detection task, and their neural responses were recorded using electroencephalogram (EEG). Second, we investigated whether ADHD symptoms are correlated with the attenuation in the neural tracking of syllables and words. The ADHD symptoms were collected from the children using the SNAP-IV Questionnaire, and the neural correlates of individual symptoms were analyzed in both the low-frequency and high-gamma bands.

## 2. Materials and methods

### 2.1. Participants

The study was approved by the Ethics Committee of Children’s Hospital of Zhejiang University (Approval number: 2020-IRBAL-023). For this study, 23 drug-naïve children (6–8 years old, mean 7.3 years old; 15 male) were recruited from Children’s Hospital of Zhejiang University. All children were referred for participation in the study by teachers or parents, and had a diagnosis of ADHD by a pediatrician. All children were right-handed, with no self-reported hearing loss or neurological disorders. Written informed consent was obtained before the experiment. Parents of these children completed the Chinese version of SNAP-IV ADHD Questionnaire (Gau et al., 2008). The SNAP-IV was designed to evaluate ADHD symptoms of children without serious comorbid conditions (Swanson et al., 2012; Zieff et al., 2022). The checklist contained three subsets of atypical behavior: inattention (INATT, nine items), hyperactivity/impulsivity (HYP/IMP, nine items), and oppositional defiant behaviors (ODD, eight items). Each item was graded from 0 to 3, with a higher score indicating an increased level of ADHD symptoms. The subscale scores were calculated by averaging scores of items within each subset.

### 2.2. Stimuli and procedures

**FIGURE 1:**
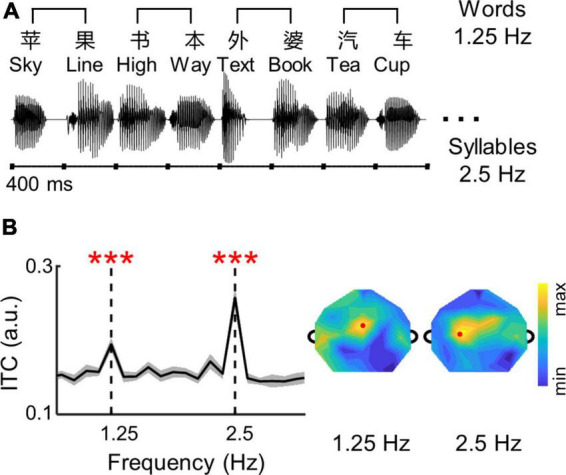
**(A)** Structure of the isochronous syllable sequences. Syllables are presented at a constant rate of 2.5 Hz. Therefore, bisyllabic words are presented at 1.25 Hz. Each trial consists of 10 bisyllabic words, lasting 8 s. The stimuli were in Chinese and English examples are shown for illustrative purposes. **(B)** The ITC values of neural responses are averaged over participants and channels. The shaded area indicates one standard error of the mean (SEM) across participants. The ITC spectrum of individual participants were shown in Supplementary Figure 3. Stars indicate significant peaks higher than their neighboring frequencies (***p < 0.001; bootstrap; FDR corrected). The topographies show distributions of ITC values. Red dots show channels with highest ITC values at 1.25 and 2.5 Hz.

The speech was created as an isochronous sequence of independent Chinese syllables, and every two syllables constituted a noun word (Figure 1A). Fifty common bisyllabic words were selected from a dataset of daily speech materials for 3–5 years old children (Liu et al., 2008), with a probe word “grandma.” Each syllable was independently synthesized using the Neospeech synthesizer (the male voice, Liang).^1^ All syllables were adjusted to the same intensity and duration (i.e., 400 ms) following the procedure in Ding et al. (2016). Each speech sequence contained 20 syllables (i.e., 10 disyllabic words), and the syllables were concatenated sequentially without any acoustic gap inserted. Therefore, the trial duration was 8 s with syllables and words presented at 2.5 and 1.25 Hz, respectively. In total, 35 trials were created. All disyllabic words were randomly distributed in the 35 trials, and each word was repeated seven times with no repeated words presented in the same trial.

For each participant, the 35 trials were presented in a random order. The participants were asked to detect whether the probe word “grandma” was presented in the speech by pressing a key (“1” for “yes” and “0” for “no”). The next trial was presented after an interval randomized between 1 and 2 s (uniform distribution) after the key press. After the experiment was finished, the SNAP-IV ADHD Questionnaire was collected from the parents of the children, and the ADHD symptom scores are shown in Supplementary Table 1.

### 2.3. EEG recording and analysis

All preprocessing and analysis in this study were performed using MATLAB (The MathWorks, Natick, MA, USA). EEG data was acquired on a 32-channel Hydrocele Geodesic Sensor Net (GSN) by Magstim EGI (Electrical Geodesic, Inc., Eugene, OR, USA) with 500 Hz sampling rate. The EEG recordings were referenced to the average of 32-channel recordings. Occasional large artifacts in EEG/EOG, that is, samples with magnitude >1 mV, were removed from the analysis (Luo and Ding, 2020). For the low-frequency band analyses, the EEG recordings were down-sampled to 120 Hz. Based on the frequency-tagging stimuli (see section “2.2. Stimuli and procedures”), the current study focused on word-rate and syllable-rate neural responses (1.25 and 2.5 Hz, respectively). To remove power-line noise (50 Hz) and slow drifts (<5 Hz), the EEG recordings were band-pass filtered between 0.6 and 20 Hz using a linear-phase finite impulse response (FIR) filter (4 s Hamming window, 6 dB attenuation at the cut-off frequencies). A linear-phase FIR filter causes a constant time delay to the input. The delay equals to N/2, where N was the window length of the filter (Oppenheim et al., 1997). The delay was compensated by removing the first N/2 samples in the filter output.

After data preprocessing, an 8-s epoch of EEG signal was obtained for each trial, and therefore the frequency resolution of the DFT analysis was 1/8 Hz. The EEG signal in the analysis window was transformed into the frequency domain using the discrete Fourier transform (DFT) without any additional smoothing window. Then, the inter-trial coherence (ITC) was calculated following (Bardouille and Ross, 2008):


$$I\,T\,C_{i}=\frac{1}{N}\,\sqrt{{\left(\sum_{n=1}^{N}s\,i\,n\,\emptyset_{i}\right)}^{2}+{\left(\sum_{n=1}^{N}c\,o\,s\,\emptyset_{i}\right)}^{2}}$$

where ∅_*i*_ was the phase at the frequency bin *i* in trial *n*. The ITC is a scalar measure bounded between 0 and 1.

### 2.4. High-gamma amplitude

Most previous studies use invasive brain imaging (e.g., electrocorticography, ECoG) to investigate neural tracking of speech in high-gamma activity (Golumbic et al., 2013; Ding et al., 2016; Akbari et al., 2019). Nevertheless, recent studies provide evidence that reliable speech-tracking neural activity in high-gamma band can also be captured using non-invasive brain imaging technologies, e.g., EEG (Synigal et al., 2020), and MEG (Kulasingham et al., 2020). Therefore, the current study further analyzed the neural tracking responses to syllables and words in the high-gamma band.

**FIGURE 2:**
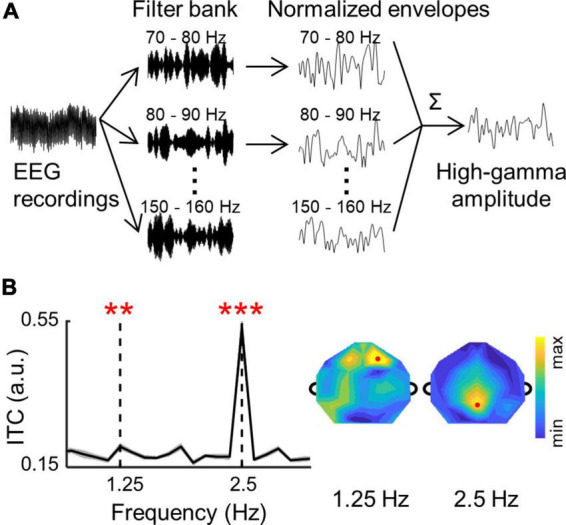
**(A)** A schematic illustration of the high-gamma amplitude analysis procedure (see section “2. Materials and methods”). **(B)** The ITC values in the high-gamma band are averaged over participants and channels. The shaded area indicates 1 SEM across participants. The ITC spectrum of individual participants were shown in Supplementary Figure 3. Stars indicate significant peaks higher than their neighboring frequencies (**p < 0.01; ***p < 0.001; bootstrap; FDR corrected). The topographies show distributions of ITC values in the high-gamma band. Red dots show channels with highest ITC values at 1.25 and 2.5 Hz.

High-gamma amplitude were extracted following the procedure in Foster et al. (2015). As shown in Figure 2A, continuous EEG recordings were filtered into a sequential of 10-Hz-width narrow band signal between 70 and 160 Hz (i.e., 70–80, 80–90,…, 150–160 Hz). To avoid artifacts of 100 and 150 Hz, surrounding filter bands were adjusted to 90–98, 102–110,…, 140–148, and 152–160 Hz. The amplitude (i.e., envelope) of each narrow band signal was extracted by *Hilbert* function in MATLAB. Then, each amplitude was normalized to its mean value in each band, and all amplitudes were averaged to obtain the high-gamma amplitude. In the frequency domain analysis, the high-gamma amplitudes were down-sampled to 60 Hz, and divided into 8-s trials. The ITC was calculated for the high-gamma amplitude following the same procedure as adopted in the analysis of the low-frequency signals.

### 2.5. Statistical test

The bootstrap significance test is a bias-corrected and accelerated procedure (Efron and Tibshirani, 1994). In the bootstrap procedure, data of all participants were resampled with replacement 10,000 times. To test the significance of the 1.25 and 2.5 Hz peaks in the ITC spectrum, the ITC at the peak frequency was compared with the mean ITC of the four neighboring frequency bins (two bins on each side, one-sided comparison). When multiple comparisons were performed, the *p*-value was further adjusted using the false discovery rate (FDR) correction (Benjamini and Hochberg, 1995). Moreover, Pearson’s correlation was calculated to quantify the relationship between neural responses and symptom scores. The significance of Pearson’s correlation coefficient for EEG channels was tested by cluster-based permutation analysis (Maris and Oostenveld, 2007). The approach was implemented using *ft_statfun_correlationT* function (based on 3,000 permutations) in the FieldTrip Matlab toolbox (Oostenveld et al., 2011). The cluster-based permutation analysis can combine spatially adjacent samples (i.e., neighboring channels) to correct for the multiple comparisons problem (Meyer et al., 2021).

### 2.6. *Post hoc* effect size calculation

A *post hoc* effect size analysis was performed to validate the appropriateness of the sample size to observe the 1.25 and 2.5 Hz responses. To simplify the analysis, we conducted a paired *t*-test to compare the ITC at the peak frequency with the mean ITC of the four neighboring frequency bins using the G*Power software (version 3.1) (Faul et al., 2007). The effect size *d* and power were reported in Supplementary Table 2, suggesting that the current study was powerful with the described sample size at the α level of 0.05.

## 3. Results

### 3.1. Behavioral performance

The experiment presented isochronous syllable sequences (Figure 1A), and the participants were asked to attend to the sequences and detect the probe word “grandma.” All participants finished the detection task, and the detection accuracy was 86.7% (SD = 6.3%). The Pearson’s correlation analysis revealed no significant correlation between the behavioral performance and ADHD symptom scores (INATT scores: *p* = 0.45, HYP/IMP scores: *p* = 0.14, ODD scores: *p* = 0.18, Pearson’s correlation, FDR corrected, Supplementary Figure 1).

### 3.2. Neural tracking of syllables and words in low-frequency activity

The EEG responses to the isochronous speech are shown in Figure 1B. The ITC spectrum was averaged over participants and EEG electrodes. Significant ITC peaks were observed at the syllable rate (2.5 Hz; *p* = 0.0001, bootstrap, FDR corrected), and at the word rate (1.25 Hz; *p* = 0.0001, bootstrap, FDR corrected). The topography of ITC showed a central distribution. All these findings suggested that low-frequency cortical activity exhibited neural tracking of syllables and words during active speech comprehension in children with ADHD, similar to healthy adults (Ding et al., 2016, 2018).

### 3.3. Neural tracking of syllables and words in the high-gamma amplitude

The neural responses to words and syllables were also analyzed in the high-gamma band (see section “2. Materials and methods” for details, Figure 2A and Supplementary Figure 4). The ITC spectrum of high-gamma amplitude is shown in Figure 2B. Similar to the results in the low-frequency band, significant ITC peaks were also observed at the syllable rate (2.5 Hz; *p* = 0.0001, bootstrap, FDR corrected), and at the word rate (1.25 Hz; *p* = 0.004, bootstrap, FDR corrected) for high-gamma amplitude. Moreover, the topography of high-gamma ITC showed a central-frontal distribution at the word rate and a central-posterior distribution at the syllable rate, which is similar to recent studies on high-gamma activity (Synigal et al., 2020; Dor-Ziderman et al., 2021). These results showed that the neural tracking of words could also be observed in the high-gamma band, and different topographic distributions suggested distinct neural source for low-frequency and high-gamma activity.

### 3.4. Relationships between ADHD scores and neural tracking responses

**FIGURE 3:**
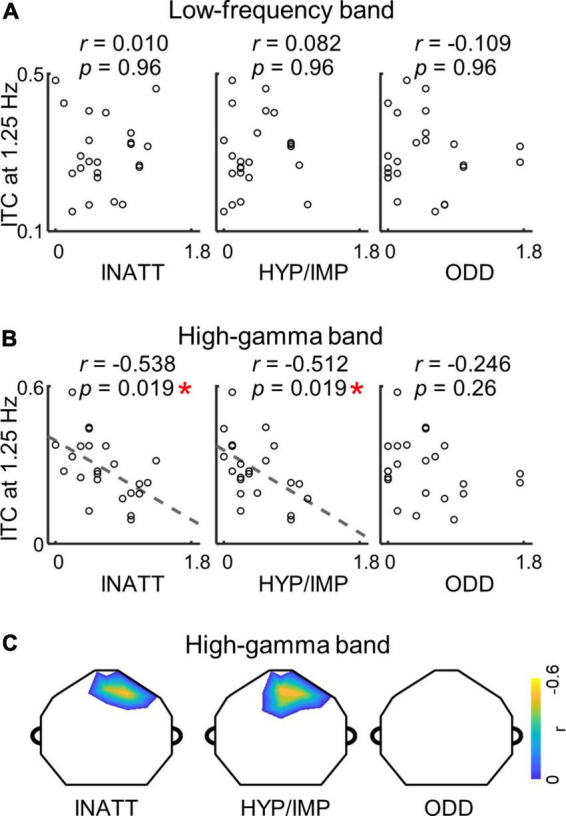
**(A)** No significant correlation is found between ADHD scores and word-rate ITC in the low-frequency band (all *p* > 0.05; Pearson’s correlation; FDR corrected). **(B)** Correlation between ADHD scores and word-rate ITC in the high-gamma band (*p < 0.05; Pearson’s correlation; FDR corrected). Each dot indicates a participant. The channel with the highest ITC values is selected for the correlation analysis. **(C)** The topographies show distributions of channels with significant correlation between ADHD scores and word-rate ITC in the high-gamma band (all *p* < 0.05; Pearson’s correlation; cluster-based permutation test).

To investigate whether cortical encoding of words and syllables was correlated with ADHD symptoms, we analyzed the relationship between low-frequency/high-gamma ITC and ADHD symptom scores. In the analysis, we selected the channel with highest averaged ITC values at the syllable or word rate, and calculated the Pearson’s correlation between the ITC values and ADHD symptom scores. At the syllable rate (Supplementary Figure 2), no signification correlation was observed between the ITC and ADHD symptom scores either in the low-frequency band (INATT scores: *p* = 0.44, HYP/IMP scores: *p* = 0.78, ODD scores: *p* = 0.44, Pearson’s correlation, FDR corrected) nor in the high-gamma band (INATT scores: *p* = 0.70, HYP/IMP scores: *p* = 0.70, ODD scores: *p* = 0.70, Pearson’s correlation, FDR corrected). At the word rate (Figure 3), no significant correlation was observed between ITC and ADHD symptom scores in the low-frequency band as well (INATT scores: *p* = 0.96, HYP/IMP scores: *p* = 0.96, ODD scores: *p* = 0.96, Pearson’s correlation, FDR corrected). In the high-gamma band, however, the word-rate ITC values was anti-correlated to the INATT scores (*r* = −0.538, *p* = 0.019, Pearson’s correlation, FDR corrected) and the HYP/IMP scores (*r* = −0.512, *p* = 0.019, Pearson’s correlation, FDR corrected) while the correlation between word-rate ITC and ODD scores was not significant (*p* = 0.26, Pearson’s correlation, FDR corrected). A data-driven analysis of individual electrodes further showed the topography of correlation in high-gamma band was located at central-frontal electrodes (Figure 3C; all *p* < 0.048, Pearson’s correlation, cluster-based permutation test), consistent with the distribution of word-rate response in the high-gamma band (topography in Figure 2). These findings revealed that word tracking activity in the high-gamma band specifically correlated to certain ADHD symptoms of inattention and hyperactivity.

## 4. Discussion

Speech perception is a complex process involving both acoustic encoding and linguistic processing, and the current study investigated which stage of speech was impaired in children with ADHD. When 6–8 years old children were engaged in a speech detection task, robust tracking responses to syllables and words were observed in both the low-frequency band and high-gamma band. We further analyzed the relationship between neural responses and ADHD symptoms. It was found that the ITC of neural response to words in the high-gamma band showed an anti-correlation with ADHD symptom scores of children, i.e., children with severer ADHD symptoms exhibited weaker word-tracking responses in the high-gamma band. These results indicated that ADHD prominently affected neural processing of linguistic information (e.g., words) during speech perception.

It is commonly observed that cortical activity tracks speech at different levels, corresponding to low-level acoustic features and high-level linguistic information (Luo and Poeppel, 2007; Ding and Simon, 2012; Brodbeck et al., 2018a,b; Keitel et al., 2018; Gao et al., 2020). Using the hierarchical auditory linguistic sequence paradigm, previous studies have found robust neural tracking responses to hierarchical speech units (e.g., syllables, words, and sentences) in adults and patients with a disorder of consciousness (Ding et al., 2016; Gui et al., 2020; Luo and Ding, 2020). Our results extended these findings and demonstrated that the hierarchical speech-tracking responses could also be observed in young children. Moreover, one line of studies has reported that cortical tracking of multiple speech features is differently modulated by selective attention (Golumbic et al., 2013; Kong et al., 2014; Ding et al., 2018; Luo and Ding, 2020; Yahav and Golumbic, 2021). Specifically, when a speech stream is presented, neural tracking of linguistic information (e.g., words, phrases, or sentences) is significantly decreased with attenuated attention, while neural tracking of acoustic features (e.g., speech envelope or rhythm) remains relatively stable. These results suggest that higher-level linguistic processing more relies on top-down attentional modulation in speech perception. Consistent with these findings, the current study found that children with severer ADHD symptoms exhibited weaker neural tracking of words (rather than syllables) in a single speech stream, indicating that the underlying attention deficits predominantly affected higher-level linguistic processing in children with ADHD.

Although cortical activity consistently tracks the temporal dynamics of speech in both low-frequency and high-gamma bands, they have non-redundant tracking properties and functional roles in speech perception (Nourski et al., 2009; Belitski et al., 2010; Golumbic et al., 2013; Mai et al., 2016; Synigal et al., 2020; Xu et al., 2023). For example, the high-gamma neural activity tracks speech with a short response latency, and remains robust for unintelligible speech (Nourski et al., 2009; Golumbic et al., 2013; Xu et al., 2023). In contrast, the low-frequency neural activity tracks speech with a long response latency, and decreases with degrading speech intelligibility (Luo and Poeppel, 2007; Xu et al., 2023). Based on these results, it has been proposed that high-gamma activity reflects automatic speech encoding in the early stage, while low-frequency activity reflects slow build-up processing in the late stage (Xu et al., 2023). Consistent with the proposal, evidence has shown that combing neural activity in the high-gamma and low-frequency bands can optimize accuracy for speech reconstruction (Golumbic et al., 2013; Akbari et al., 2019; Synigal et al., 2020). Here, our results showed that ADHD symptoms correlate to high-gamma responses but not to low-frequency responses, further supporting the previous suggestion that these two frequency bands represent systematically different mechanism and function for speech processing. Furthermore, evidence has shown that low-frequency and high-gamma responses exhibit distinct neural sources during speech perception (Crone et al., 2001; Edwards et al., 2009; Synigal et al., 2020). Specifically, low-frequency activity is distributed across temporal, frontal, and parietal lobes (Towle et al., 2008; Sinai et al., 2009), while high-gamma activity is mainly localized to the superior temporal gyrus (Crone et al., 2006; Golumbic et al., 2013). Consistent with these findings, the topographical plots in the current study also displayed different spatial distributions between low-frequency and high-gamma responses (Figure 1 vs. Figure 2).

Previous studies on healthy individuals have demonstrated that high-gamma neural activity is associated with processes of attention (Ray et al., 2008; Akimoto et al., 2014; Fiebelkorn and Kastner, 2019), working memory (Brovelli et al., 2005; Jensen et al., 2007; Wilsch and Obleser, 2016), and speech perception (Kubanek et al., 2013; Ding et al., 2016; Kulasingham et al., 2020). Therefore, it seems plausible to assume that the atypical cognitive function of ADHD children could stem from altered high-gamma neural activity (Herrmann and Demiralp, 2005). Previous EEG studies have shown evidence for significant differences between ADHD children and healthy controls in both resting-state and task-related neural activity in the gamma band (Lenz et al., 2008; Barry et al., 2010). A recent MEG study has also demonstrated that resting-state high-gamma power can predict cognitive performance and emotional state in ADHD children (Brovelli et al., 2005). Our results extended these findings, and demonstrated that the high-gamma neural response to words during speech perception is correlated with atypical symptoms in ADHD children. Since the cognitive function of attention and working memory (WM) is universally impaired in ADHD individuals (Hale et al., 2005, 2007; Halder and Kotnala, 2018; Ramos et al., 2020), the neural correlates of ADHD symptoms might reflect the influence of attention or WM impairments on linguistic processing in children with ADHD.

There are also limitations in the current study. It should be noted that the children who participated in the current study only had mild ADHD symptoms since we found children with severe ADHD were hard to remain stationary and complete all experimental procedures during EEG recordings. Although the current studies exhibited that ADHD symptoms were only correlated with neural impairment in linguistic processing, we cannot exclude the possibility that severer ADHD or different ADHD subtypes might further influence acoustic processing in speech perception. Future efforts are encouraged to adopt a more fine-grained paradigm and investigate how speech processing is impaired in children with severe ADHD or with different ADHD subtypes. Moreover, neither ADHD symptoms nor neural responses are correlated with behavioral performance in the current study (see Supplementary Figure 1). This is probably because word detection in a quiet listening condition is a relatively easy task (Luo and Ding, 2020; Liu et al., 2022), and most children achieved high performance (86.7 ± 6.3%) in the current study. Therefore, the measure of detection performance might not be sensitive enough to detect language impairment in children with ADHD. Future work could use the paradigm in more challenging or sophisticated settings (e.g., complex auditory scenes and difficult speech materials) to investigate the underlying neural correlates of individual behavioral performance in children with ADHD. Finally, the current study is also limited by its small sample size and lack of a clinical control group, which should be addressed by future studies. Nevertheless, it provides initial evidence for the relationship between task-related high-gamma activity and ADHD symptoms. Due to the evolving understanding in both the anatomical and functional level of high-gamma activity, focusing future studies on the high-gamma band bears the potential for better understanding of ADHD, and developing new tools for evaluation and therapy.

In sum, the current study demonstrates that cortical activity can track different levels of speech units (e.g., syllables and words) during speech perception in young children with ADHD, and ADHD predominantly impairs cortical encoding of linguistic information (e.g., words) in speech perception.

## Data availability statement

The datasets presented in this study can be found in online repositories. The names of the repository/repositories and accession number(s) can be found in the article/Supplementary material.

## Ethics statement

The studies involving human participants were reviewed and approved by the Ethics Committee of Children’s Hospital of Zhejiang University. Written informed consent to participate in this study was provided by the participants’ legal guardian/next of kin.

## Author contributions

CL designed the research. YY and XZ performed the research. CL, JF, YL, XZ, and YG analyzed the data. CL and YG wrote the manuscript. All authors contributed to the article and approved the submitted version.

## Footnotes

1
http://www.neospeech.com/
