# Supplementary material for: Compromised word-level neural tracking in the high-gamma band for children with attention deficit hyperactivity disorder
Source: Front Hum Neurosci. 2023 May 5;17:1174720. doi: 10.3389/fnhum.2023.1174720 (PMC10196181; doi:10.3389/fnhum.2023.1174720)
Supplement: Supplementary file 1 [file Data_Sheet_1.docx]

**Supplementary Table 1** Demographic and SNAP-IV scores of participants. The SNAP-IV contained three subsets of atypical behaviour: inattention (INATT, 9 items), hyperactivity/impulsivity (HYP/IMP, 9 items), and oppositional defiant behaviours (ODD, 8 items). Each item was graded from 0 to 3, with a higher score indicating an increased level of ADHD symptoms, and the subscale scores were calculated by averaging scores of items within each subset.

| **Participant No.** | **Age** | **Sex** | **INATT** | **HYP/IMP** | **ODD** |
| --- | --- | --- | --- | --- | --- |
| 1 | 7Y2M | male | 0.6 | 0.3 | 1.8 |
| 2 | 7Y | male | 1 | 0.4 | 0.5 |
| 3 | 7Y7M | female | 1.1 | 1 | 1 |
| 4 | 6Y7M | female | 0.8 | 0.2 | 0.1 |
| 5 | 7Y1M | female | 0.4 | 0 | 0.5 |
| 6 | 6Y5M | male | 0.3 | 0.1 | 0 |
| 7 | 7Y1M | male | 0.6 | 0.3 | 0 |
| 8 | 6Y9M | male | 0.4 | 0.1 | 0.8 |
| 9 | 6Y7M | male | 0.2 | 0.1 | 0.1 |
| 10 | 6Y9M | male | 0.2 | 0 | 0.6 |
| 11 | 8Y | male | 0.9 | 1.1 | 0.8 |
| 12 | 8Y1M | male | 0.4 | 0.2 | 0.1 |
| 13 | 6Y8M | female | 1.1 | 0.2 | 1 |
| 14 | 7Y3M | male | 0 | 0.1 | 0.3 |
| 15 | 6Y9M | male | 0.7 | 0.7 | 0.1 |
| 16 | 8Y3M | female | 0.4 | 0.6 | 0.5 |
| 17 | 8Y3M | female | 1.2 | 0.9 | 1.8 |
| 18 | 6Y8M | female | 0.6 | 0.2 | 0 |
| 19 | 8Y11M | male | 1 | 0.9 | 0.4 |
| 20 | 7Y9M | male | 1.3 | 0.6 | 0.5 |
| 21 | 7Y9M | female | 0.1 | 0.1 | 0 |
| 22 | 7Y4M | female | 0.3 | 0.2 | 0 |
| 23 | 6Y | male | 1 | 0.9 | 0.9 |

Y, year; M, month.

**Supplementary Table 2** The effect size and power of ITC at 1.25 and 2.5 Hz.

|  | ***d*** | **Power** |
| --- | --- | --- |
| **Low-frequency band** |  |  |
| 1.25 Hz | 0.95 | 0.99 |
| 2.5 Hz | 1.34 | 0.99 |
| **High-gamma band** |  |  |
| 1.25 Hz | 0.49 | 0.73 |
| 2.5 Hz | 1.31 | 0.99 |


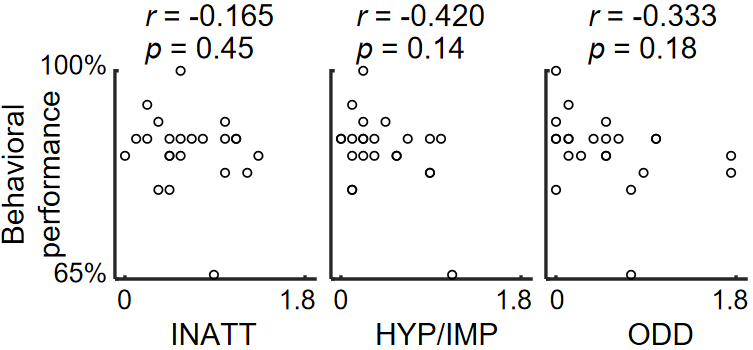


**Supplementary Figure 1.** No correlation was found between behavioral performance and ADHD symptom scores.


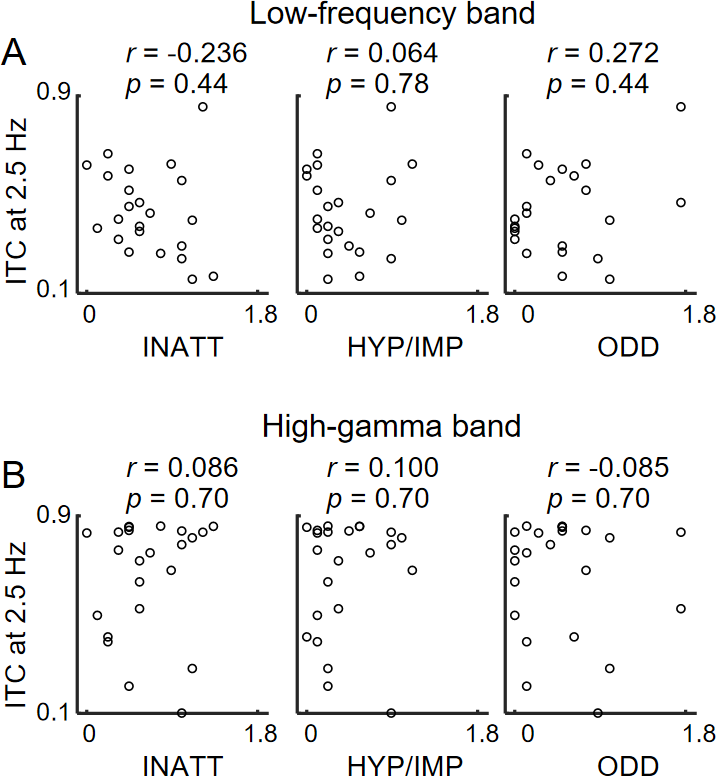


**Supplementary Figure 2**. No correlation is found between ADHD scores and syllable-rate ITC in the low-frequency band (**A**) nor in the high-gamma band (**B**). Each dot indicates a participant. The channel with highest ITC values is selected in the correlation analysis.


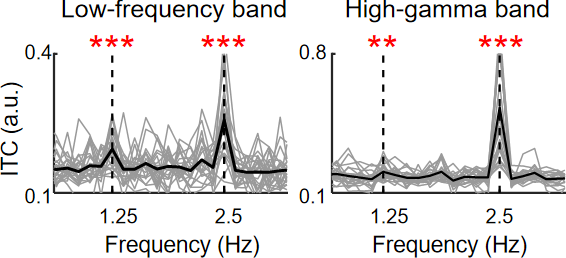


**Supplementary Figure 3**. The ITC spectrum of individual participants in the low-frequency band (left) and in the high-gamma band (right). Black lines plot ITC averaged over participants. Stars indicate significant peaks higher than their neighboring frequencies (**, p < 0.01; ***, p < 0.001; bootstrap; FDR corrected).


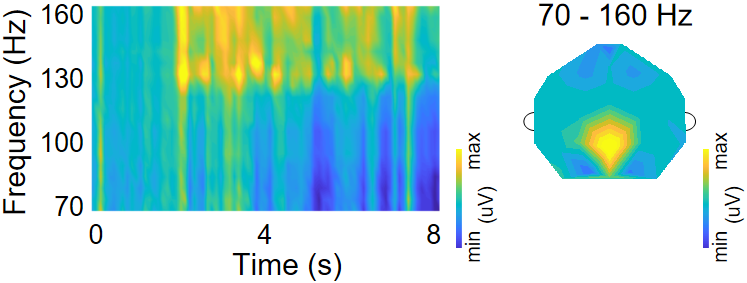


**Supplementary Figure 4.** Spectrum of the neural response to sound stimuli (left) and topography of high-gamma response (right). The response spectrum is averaged over participants and electrodes. The response topography shows a central-posterior distribution.
